# Comparative evaluation of crestal bone loss around dental implants following platelet concentrates and beta TCP bone graft

**DOI:** 10.6026/973206300214158

**Published:** 2025-11-15

**Authors:** Kumar Saurabh, Shailendra Kumar Dubey, Tripta Agarwal, Vidisha Gaur, Neeta Sinha, Sarita Singh

**Affiliations:** 1 Department of Prosthodontics and Crown & Bridge, Patna Dental College & Hospital, Patna, Bihar, India; 2 Department of Dentistry, Prosthodontics and Crown & Bridge, Netaji Subhas Medical College & Hospital, Bihta, Patna, Bihar, India; 3 Department of Dentistry, Nandkumar Singh Chouhan Government Medical College, Khandwa, Madhya Pradesh, India; 4 Department of Periodontics and Implantalogy, Maulana Azad Institute of Dental Sciences, Delhi, India; 5 Department of Prosthodontic and Crown & Bridge, Buddha Institute of Dental Sciences and Hospital, Patna, Bihar, India

**Keywords:** Bone graft, bone loss, platelet concentrates, PRP, TCP

## Abstract

The degree of crestal bone loss surrounding dental implants (mare than 0.2 mm) significantly affects their success rate. So, the crestal bone loss surrounding dental implants following the implantation of platelet concentrates and beta-tricalcium phosphate bone grafts was compared. In 100 subjects, two implants each were placed in edentulous spaces where one implant site of Group I was filled with β-Tricalcium Phosphate Bone Graft along (β-TCP) with platelet-rich plasma (PRP) and for Group II only β-Tricalcium Phosphate Bone Graft along (β-TCP) was placed in posterior edentulous area and followed for 3, 6 and 9 months using periapical radiographs. In both groups, the highest mean crestal bone loss was highest on the lingual side of the implant. Adding PRP (platelet rich plasma) to beta tricalcium phosphate helps in reduced bone loss in dental implants.

## Background:

Replacement of lost teeth using dental implants has resulted in a vital revolution linked with modern clinical dentistry. However, dental implants have been linked with surrounding bone loss in nearly 5-105 subjects where dental implants are placed. An implant is considered a failure when it depicts peri-implant bone loss of >1 mm, is mobile, or lost after a year of its placement. Peri-implantitis can lead to bone loss around the implant and further implant loss [1]. Peri-implantitis is a site-specific infectious and inflammatory condition of soft tissue and results in bone loss around a functional and osseointegrated dental implant. The long-term implant success is largely governed by bony support preservation around dental implants which is assessed radiographically [2]. Bone substitutes are often utilized to restore and fill the bone deficiency in order to treat peri-implantitis in conjunction with conservative and surgical therapy. Among the most popular bone grafts and replacements is TCP (tricalcium phosphate), which is recognized for use in the treatment of peri-implantitis and is frequently used in dental implants and after tooth extraction to fill up bone abnormalities. Due to their degrading qualities, beta tricalcium phosphate (β TCP) and hydroxyapatite and β TCP have also been employed since they do not cause harmful cellular responses and are eventually replaced by bone or undergo osseointegration [3]. Marginal bone remodeling of <2mm in the first year of implant placement and <0.2 mm every year following. These alterations are usually linked to the use of implants having a conventional machined surface and a neck design. However, in recent times, literature data has reported that implants with micro thread neck design and the rough surface can improve the stabilization and preservation of the crestal bone [4]. Therefore, it is of interest to comparatively evaluate the crestal bone loss around the dental implants after placement of platelet concentrates and beta-Tricalcium Phosphate bone graft.

## Materials and Methods:

The present prospective clinical study was directed to comparatively evaluate the crestal bone loss around the dental implants after placement of platelet concentrates and beta-tricalcium Phosphate bone graft. Verbal and written informed consent was taken from all the subjects before study participation. Study was performed at Department of Prosthodontics, Crown & Bridge, Buddha institute of Dental Sciences & Hospital, Patna, Bihar after Ethical Clearance. [BIDSH/IEC/13/2022] in the period between May 2022 to December- 2024. The study assessed 100 subjects who needed the replacement of two teeth using a dental implant-supported prosthesis in the posterior mandibular region and were aged 20-60 years. The inclusion criteria for the study were subjects with good oral hygiene, adjacent teeth to edentulous space with no periapical lesion and periodontally healthy status, non-smokers and subjects smoking <3 Cigarettes/day. The exclusion criteria for the study were subjects with inadequate bone volume, non-treated periodontal disease, oral par function, long-term oral medications, breastfeeding, pregnant females, alcoholic, tobacco or betel nut chewing, smokers and systemically unhealthy subjects. The study included 100 subjects concerning the inclusion criteria. Each subject was given one implant (Nobel Biocare) in a posterior mandibular region filled with β-Tricalcium Phosphate Bone Graft along (β-TCP) and PRP (Platelet-Rich Plasma) as Group I and another edentulous space was given one implant (Nobel Biocare) in a posterior mandibular region filled with only β-Tricalcium Phosphate Bone Graft that comprised Group II. Before implant placement, platelet concentrates (platelet-rich fibrin) were procured by centrifugation of the blood samples from autologous blood of the subjects collected in aseptic condition in 10 ml test tubes without any anti-coagulants. The blood was centrifuged for 10 minutes at 3000 rpm. Centrifuged blood had many parts rich in platelets known as buffy coats that were collected, cut and mixed with particulate β TCP.

Fibrin-rich parts were pressed to obtain the PRP membrane. For implant placement, a full-thickness mucoperiosteal flap was raised under local anesthesia following a crestal incision in the edentulous area. This was followed by the placement of dental implants and graft materials following the group where the treated site was allocated and suturing was done for the flap. Subjects were given postoperative instruction and advised 0.12% chlorhexidine rinse twice daily for 10 days and sutures were removed after 15 days. All implants were submerged to the level where the outer edge reached marginal bone level allowing the cover screw apex at bone crest level during healing. Digital periapical radiographs were taken postoperatively to assess the first contact level of the implant to crestal bone from the implant top along the body/collar surface in each implant on the buccal, lingual, mesial and distal side. These assessments served as baseline reference values to assess future bone loss. After three months of implant placement, second-stage surgery was done and healing abutments were placed over dental implants followed by measurement of crestal bone levels on the buccal, lingual, mesial and distal side of each implant using periapical radiography. Crestal bone loss calculation was done by subtraction of baseline reference bone levels from present bone levels which depicted 3 months of bone loss. After 2 weeks postoperative, impressions were made to healing abutment surgery connection. A permanent prosthesis was given after two weeks of impression. Digital periapical radiographs of the implant site were done at 3 months, 6 months and 9 months after placement of dental implants. The data collected were expressed as frequency and percentage and mean and standard deviation which was assessed using SPSS (Statistical Package for Scientific Studies) software version 20 (IBM, Armonk, NY, USA) for statistical analysis.

## Results:

The study results depicted no significant difference in demographics in the two groups where 100 subjects comprising 50 females and 50 males were given 200 dental implants. Mean values of crestal bone loss at various time intervals in Group I and II subjects are summarized in Tables 1 (see PDF). The mean bone loss around the perimeter of Group I and II implants after 3 months was 1.21 and 1.59 mm was 1.66 and 2.0 mm after 6 months of implant placement and after 9 months of implant placement, it was 2.21 and 2.73 mm respectively. Average crestal bone loss in implants from Group I after 3 months of implant placement was 1.0±0.36, 1.18±0.38, 1.28±0.40 and 1.38±0.39 mm on mesial, distal, buccal and lingual side respectively. After 6 months, crestal bone loss was 1.38±0.40, 1.68±0.41, 1.78±0.43 and 1.79±0.42mm respectively. At 9 months of implant placement, crestal bone loss was 2.0±0.4, 2.19±0.46, 2.29±0.48 and 2.38±0.47 mm on mesial, distal, buccal and lingual sides respectively (Table 1 - see PDF).

## Discussion:

For dental implants, success is largely governed by implant design which has been widely established. PRP is a blood derivative having a high concentration of platelets. In clinical dentistry, various platelet concentrates have been used including PRP in the management of musculoskeletal diseases and reported efficacy in regeneration of ligaments, tendons, muscle, bone and cartilage. The study results showed that PRP depicts a radiographic maturation rate of 1.6-2 times higher than without PRP at 6 months follow-up with greater bone density. Since this study, PRP use has been explored widely as an augmentation procedure of dental implants as suggested by Roca-Millan *et al.* in 2022, [3] and Lu *et al.* in 2021 [4]. The crest module in an implant is a transosteal part of the metal dental implant creating a transition zone to load bearing implant part and is designed to hold the components of implant prosthesis in place as reported by Cheah *et al.* in 2021 [5]. The implant collar is designed to decrease plaque accumulation and is a polished surface with different lengths. The height of the tissue above the dental implant is generally 2.5mm and brush bristles are unable to reach beyond 1mm hence; smooth collars can result in bone loss. Crestal bone is strongest against compressive forces and weakest against shear forces. Smooth collar led to shear forces on crestal bone and resultant bone loss can be attributed to mechanical stimulation lack in crestal area. A crest module angled >20 degrees with surface texture increasing bone contact height can result in tensile and compressive components, minimizing crestal bone loss.

The results of the study were also to the study by Li *et al.* in 2021 [6] where three implants were placed immediately following the extraction of a single-rooted tooth using human mineralized cancellous bone complement which was also placed in the socket preserved using human mineralized cancellous bone allograft. Radiographic assessment after 4 months of implant placement showed mean bone loss of 0.48 ± 0.53 mm and 0.51 ± 0.41 mm at distal and mesial side. Another study by Jalaluddin *et al.* in 2024 [7] used biphasic calcium phosphate ceramic granules in 12 patients to prevent bone loss and after 5 years, 3D CBCT (cone-beam computed tomography), clinical and minimum control were done. The authors reported good clinical implant stability with no inflammation sign. A study by Abulhassan *et al.* in 2024 [8] reported flapless crestal sinus augmentation surgery using BMP-2-loaded Bio-Oss collagen, with nonfunctional implants immediately loaded after surgery. Bone height was measured with preoperative and postoperative CBCT (cone beam computed tomography). At sinus graft sites, bone density, marginal bone loss and 3 months implant stability were assessed using Periostat and postoperative CBCT scans. Results showed good implant stability and high bone density level depicting minimum marginal bone loss after 37.3 months. This was similar to the present study where the difference in crestal bone loss at 3 months was statistically non-significant. Lack of positive stimulation and surgical trauma owing to occlusal forces can also result in crestal bone loss around dental implants. The results of the present study reported that after 6 months of implant placement, overall crestal bone loss in Groups I and II was 1.66 and 2.01 mm respectively. Bone loss difference was statistically non-significant with a mean difference of 0.31. After 9 months of implant placement, a significant difference was seen in crestal bone loss in Group I and II with p<0.05. This was in agreement with the studies of Orhan *et al.* in 2024 [9] where mean peri-implant bone loss at 3 years follow-up was 1.00±0.1 mm for the group without alveolar preservation and 1.00±0.2 mm in the socket preservation group using xenograft. Also, the authors reported no significant difference in marginal bone loss in the two groups at 1, 2 and 3 years. Hence, the addition of PRP in TCP around implants helps in excessive bone loss prevention compared to TCP alone around dental implants.

## Conclusion:

Crestal bone loss around dental implants along with tricalcium phosphate bone graft and PRP result in lesser bone loss compared to implants placed with tricalcium phosphate bone graft alone. However, it can be concluded that various bone substitutes are presently applied in clinical dentistry. However, none of the materials meet the criteria of an ideal implant bone graft. Ideally, to attain good osseointegration, bone implants must have four elements including osteoinductive factors, osteogenic cells, osteoconductive matrix and structural integrity.
